# Genome-wide DNA methylation signatures to predict pathologic complete response from combined neoadjuvant chemotherapy with bevacizumab in breast cancer

**DOI:** 10.1371/journal.pone.0230248

**Published:** 2020-04-16

**Authors:** Ping-Ching Hsu, Susan A. Kadlubar, Eric R. Siegel, Lora J. Rogers, Valentina K. Todorova, L. Joseph Su, Issam Makhoul

**Affiliations:** 1 Department of Environmental and Occupational Health, College of Public Health, University of Arkansas for Medical Sciences, Little Rock, Arkansas, United States of America; 2 Department of Biostatistics, Colleges of Medicine and of Public Health, University of Arkansas for Medical Sciences, Little Rock, Arkansas, United States of America; 3 Department of Epidemiology, College of Public Health, University of Arkansas for Medical Sciences, Little Rock, Arkansas, United States of America; 4 Department of Internal Medicine, College of Medicine, University of Arkansas for Medical Sciences, Little Rock, Arkansas, United States of America; Chinese University of Hong Kong, HONG KONG

## Abstract

Neoadjuvant chemotherapy is given before surgery to patients with primarily unresectable, advanced-stage cancers to render the tumor resectable, and to facilitate breast conservation. Previously, we have reported a prospective phase II trial for women with stage IIA-B/IIIA-B-C breast cancer with improved pathologic complete response (pCR) when using bevacizumab in the neoadjuvant setting. Chemotherapy agents are given intravenously during multiple cycles of systemic treatments. However, the effect of the treatment is only evaluated upon the completion of therapy. Here, data from a clinical trial of 40 breast cancer patients with very aggressive disease and poor prognosis were studied aiming to identify epigenetic signatures in blood-derived DNA at baseline as potential non-invasive markers to predict pCR and to determine if treatment-related changes in epigenetic profiles reflect responsiveness to therapy. We performed genome-wide DNA methylation profiling using blood-derived DNA, and found that pre-treatment methylation status of *BRD9* was predictive of responsiveness to therapy. Post-treatment global methylation differences were also observed between responders and non-responders. Most differentially methylated (DM) CpGs were located in promoter CpG-island regions for responders and in the open-sea region for non-responders. In responders, *DNMT3B* was hypomethylated while most of the other genes were hypermethylated after 4 cycles of treatment. Hypomethylation of *DNMT3B* could potentially lead to the increased methylation of oncogenes and genes responsible for cell growth and proliferation, facilitating responsiveness to the therapy. These results support the possible development of *BRD9* as a biomarker for treatment selection before neoadjuvant therapy with chemotherapy and bevacizumab, and indicate *DNMT3B* as a potential target to improve clinical response. Further prospective validation of these findings is warranted.

**Trial registration:**

ClinicalTrials.gov Identifier: NCT00203502.

## Introduction

In oncology, identification of predictive and prognostic biomarkers of treatment response is an area of intensive research. Genomic profiling has revealed tumor mutations and genetic variants that guide therapeutic decision making. In addition to tumor characteristics, host genetic variability also plays a role in treatment efficacy, and incorporation of genomic information into clinical decision making is a goal of precision medicine.

Neoadjuvant chemotherapy (NCT) is widely used in breast cancer before surgery to decrease tumor volume and facilitate surgical resection. Reduction of tumor volume in many cases allows breast-conserving surgery and the avoidance of mastectomy. In some cases, NCT results in the complete disappearance of the tumor prior to surgery, generating a pathological Complete Response (pCR). Either pCR or marked tumor reduction represent a net benefit to the patient. However, sometimes NCT has little direct effect on the tumor, which means the patient then endures ineffective treatment that can have long-lasting, and potentially irreversible, adverse effects. Clearly, it would be clinically useful in patient management to have a biomarker-based assay that can predict how well or how poorly the breast cancer patient’s tumor responds to NC.

Genetic-variation studies have primarily been focused on tumor somatic mutations or on germline single-nucleotide polymorphisms (SNPs). However, it is becoming increasingly appreciated that epigenetic modifications controlling the expression of critical genes also contribute to therapeutic response. The most common epigenetic modification, and the one that has received the most attention to date, is cytosine methylation at cytosine-guanine dinucleotide (“CpG”) sites or islands along the DNA sequence. Differences in CpG-island methylation status between different subjects have been shown to be associated with phenotype differences that include both a subject’s susceptibility to disease and a disease’s susceptibility to treatment. Likewise, changes in CpG methylation over time within the same subject have been associated with normal life-cycle processes ranging from embryogenesis to aging and senescence. Changes in CpG-island methylation have also been related to pathological processes such as carcinogenesis, responsiveness to starvation, gluttony, dietary imbalances, and exposures to pollutants, toxins, phytochemicals, and chemotherapy agents. Therefore, incorporation of differential CpG-island methylation detection into a biomarker-based assay has the potential to improve the prediction of response to NC, and thus refine precision-medicine practices.

Here, we report the results of a correlative study of CpG methylation in prospectively enrolled breast cancer patients that received NCT for their disease. Our purpose was two-fold. First, we sought to assess whether the methylation status of certain CpG-islands at baseline (before NCT started) was associated with achievement of pCR six months after NCT began. Second, we sought to study whether and/or how the methylation status of CpG-islands changes in response to treatment with NCT, and whether the pattern of change in CpG-methylation status was different among subjects who attained pCR compared to subjects who did not.

## Material and methods

### Study design & study participants

Detailed study methods for subject accrual have been described previously [1, 2]. Briefly, the study included 40 women with very aggressive disease and poor prognosis enrolled after providing written informed consent in NCT00203502 (ClinicalTrials.gov), a prospective single-arm, single-institution phase II study approved by the Institutional Review Board (IRB) of the University of Arkansas for Medical Sciences between September 2005 and January 2008. Enrolled participants consisted of women older than 18 years with histologically confirmed stage II or III breast cancer, who did not have inflammatory BC (T4d) or any recent arterial thromboembolic events, uncontrolled hypertension, recent major surgery, or baseline left ventricular ejection fraction (LVEF) <50%. The participants were offered chemotherapy combination with docetaxel (75 mg/m2), cyclophosphamide (500 mg/m2), and bevacizumab (15 mg/kg) every 3 weeks×4 cycles followed by doxorubicin (60 mg/m2) every 3 weeks×4 cycles before undergoing surgery for their cancer. After surgery, patients were evaluated for pCR as the primary clinical endpoint, defined as the absence of invasive cancer in the breast specimen. Of the 40 patients enrolled, one withdrew consent and one did not undergo surgery after NCT but she was included in the intent to treat analysis. Sixteen (41%) of the 39 evaluable subjects attained pCR, significantly higher than the null-hypothesis rate of 25% (P = 0.0204). We have previously reported that the planned sample size of 40 subjects gave NCT00203502 sufficient power to detect a hypothesized increase of 20 percentage points in pCR [2]. For this report, we undertook retrospective power analyses of post-treatment changes in methylation status detectable at FDR-adjusted *p* < 0.05 in the responder and non-responder subgroups. The results of that retrospective power analysis appear below, and show that the subgroups had adequate sample sizes for the planned methylation analysis.

### Sample collection, storage, and DNA isolation

Blood samples were collected from patients at baseline, after 4 cycles of docetaxel, cyclophosphamide and bevacizumab (DCB), and just before infusion at each of the 8 preoperative chemotherapy cycles. Blood components were separated, aliquotted into cryovials, and stored at -80°C for later analyses. Genomic DNA was extracted from buffy coats of the blood samples collected using Maxwell (Promega, Fitchburg, WI, USA). Concentration and quality of the extracted genomic DNA (gDNA) was measured using the Nanodrop 8000 instrument (Thermo Scientific, Waltham, MA, USA).

### Infinium methylation EPIC beadChip analysis

Following bisulfite treatment of 1 μg genomic DNA using the EZ DNA Methylation kit (Zymo Research, Irvine, CA), the bisulfite-converted DNA was hybridized onto the Infinium Methylation EPIC BeadChip (Illumina, San Diego, CA), following the Illumina Infinium HD Methylation protocol in the Genomics Core Facility at UAMS. The Methylation EPIC BeadChip covers over 850,000 CpG sites, and has increased genome coverage of regulatory regions and higher reproducibility and reliability compared to previous versions [3]. Whole genome amplification, hybridization, staining and scanning steps for all samples were performed, the Illumina iScan SQ scanner was used to create images of the single arrays, and the intensities of the images were extracted using the Methylation module (v.1.9.0) of the GenomeStudio (v.2011.1) software (Illumina). Raw intensity data as IDAT files were imported into GenomeStudio for the computation of detection *p*-value of the probes, and all further steps including data import, normalization, filtering and analyses were performed using the methylation pipeline in Partek Genomics Suite^™^ 6.6 (Partek Inc., St. Louis, MO).

### Methylation data analysis

The data was normalized using the Sub set-quantile Within Array Normalization (SWAN)[4]. For analysis, probes were excluded if detection *p* values were > 0.01 (n = 130), or if SNPs were present in the target CpG based on NCBI dbSNP Build 138 (n = 59). Probes on the X and Y chromosomes (n = 19,572), and probes with polymorphic targets & cross-hybridization potential [5] (n = 318,809) were also excluded. The final dataset contained 527,348 probes across all samples. Percent methylation values for each CpG site (β-values) and logit-transformed ratios of methylated to unmethylated probe intensities (M-values) were extracted for further analysis [6]. Since race plays an important role in genome-wide DNA methylation [5], data from one Asian and one Hispanic participant was excluded from the analysis.

For pattern identification in DNA methylation, unsupervised analysis including unsupervised hierarchical clustering [7] and Principal Component Analysis (PCA) were used. *T*-tests and chi-square (*X*^*2*^) tests were performed to evaluate differences between non-responders and responders. Analysis of variance (ANOVA) adjusting for race, tumor type, and estrogen receptor (ER) status with Fisher's Least Significant Difference contrast method were used to assess the differentially methylated (DM) CpG sites univariately for their potential to predict the subsequent response to therapy. The resulting P-values were adjusted for multiple testing with the false discovery-rate (FDR) procedure of Benjamini and Hochberg [8], and significance was granted with FDR *P*-value <0.05 to identify significantly differential DNA methylation. In subgroup analysis, we examined post-treatment changes from baseline within each responder group, again using ANOVA adjusting for race, tumor type, and estrogen receptor (ER) status with Fisher's Least Significant Difference contrast method. Classical Receiver Operating Characteristic (ROC) curve analysis was used to evaluate the performance of a single CpG site as a biomarker.

To determine whether the responders and non-responders had adequate sample sizes for the subgroup analysis of post-treatment change in methylation, a retrospective power analysis was conducted using the FDR-control method of Jung *et al*. [9]. We define a truly positive CpG site to be one that shows a standardized change of ≥1.5 SD in post-treatment methylation level compared to baseline. To calculate power retrospectively for multiple 2-sample t-tests with FDR set equal to 5%, we hypothesis that 1.00% (5,274) of the 527,348 CpG sites in the final data set satisfy the ≥1.5-SD criterion of being truly positive. Under this hypothesis, a sample size of N = 16 responders provides the multiple 2-sample t-tests at FDR = 5% with 89.77% power per test to detect a truly positive CpG site, and yields a binomial expected value ±1 SD of 4,734 ±22.0 positive test results (true discoveries) from the 5,274 CpG sites hypothesized to be truly positive. Under the same hypothesis, a sample size of N = 21 non-responders provides the multiple 2-sample t-tests at FDR = 5% with 99.01% power per test to detect a truly positive CpG site, and yields a binomial expected value ±1 SD of 5,222 ±7.2 positive test results (true discoveries) from the 5,274 CpG sites hypothesized to be truly positive. These retrospective power calculations demonstrate that both the responders and the non-responders had sample sizes large enough to power adequately the subgroup analysis of methylation response to treatment.

### Pathway analysis

The genes corresponding to the significant loci were projected onto knowledge-based networks for their potential biological implications using Ingenuity Pathway Analysis (IPA^®^, www.qiagen.com/ingenuity). IPA employs information obtained from the literature to assemble and extrapolate known interactions, signaling, as well as the relationships between the molecules. The analysis here was restricted to genes or molecules that have been observed directly in human breast or immune cell lines in the literature. Network analysis was generated *de novo* based on the mapped CpG sites to explore potential molecular events and mechanisms impacted by the DM CpGs. The P-score [computed as–log_10_(*P*-value)] reflected the probability of the identified genes in a list of biological functions stored in the IPA knowledge base.

## Results

### Demographics of the study participants

Samples for CpG methylation analysis were available for 37 patients; their demographics and disease characteristics are shown in Table 1. The two groups differed significantly at *p* < 0.05 in race (African American, AA; and European American, EA) and estrogen receptor (ER) status. Although differences in the type of tumor did not reach statistical significance (*p* = 0.07), a tendency was observed for most of the lobular and poorly differentiated breast cancer patients to be non-responders. Thus, race, ER status, and tumor type were included as covariates to adjust for in the subsequent analysis.

**Table 1:** Demographics and disease characteristics of study participants.

Total 37	21 Non-responders (0)	16 Responders (1)	*p*
Race			*0*.*02*
Black	3	9	
White	18	7	
Age	46.4 ± 12.9	44.6 ± 12.9	0.68
Tumor type			0.07
Ductal	13	15	
Lobular	5	1	
Poorly diff	3	0	
ER			*0*.*03*
Positive	14	4	
Negative	7	12	
PR			0.63
Positive	8	4	
Negative	13	12	
HER2			1.00
Positive	5	3	
Negative	16	13	
Triple negative			0.32
Yes	6	8	
No	15	8	
Tumor grade			0.70
I	1	1	
II	8	4	
III	12	11	
Both cycles complete			0.66
Yes	16	14	
No	5	2	
FU in years (yrs)	2.8 ± 1.4	3.5 ± 0.9	0.13
Tumor 1st diameter (cm)	6.2 ± 2.8	4.9 ± 1.9	0.13
Tumor 2nd diameter (cm)	6 ± 2.8	4.9 ± 2.3	0.29
Tumor max diameter (cm)	6.5 ± 2.8	5.1 ± 2.0	0.11

^a^*p*-values represent differences in non-responders vs. responders for each characteristic. Continuous variables were evaluated by two-sample t-tests, and chi square (*X*^2^) tests were used to investigate the differences in distributions of categorical variables from non-responders to responders.

^b^One Hispanic and one Asian (both non-responders) were excluded from the Race comparison.

### DM CpG sites at baseline predictive of pathologic response

**Fig 1:**
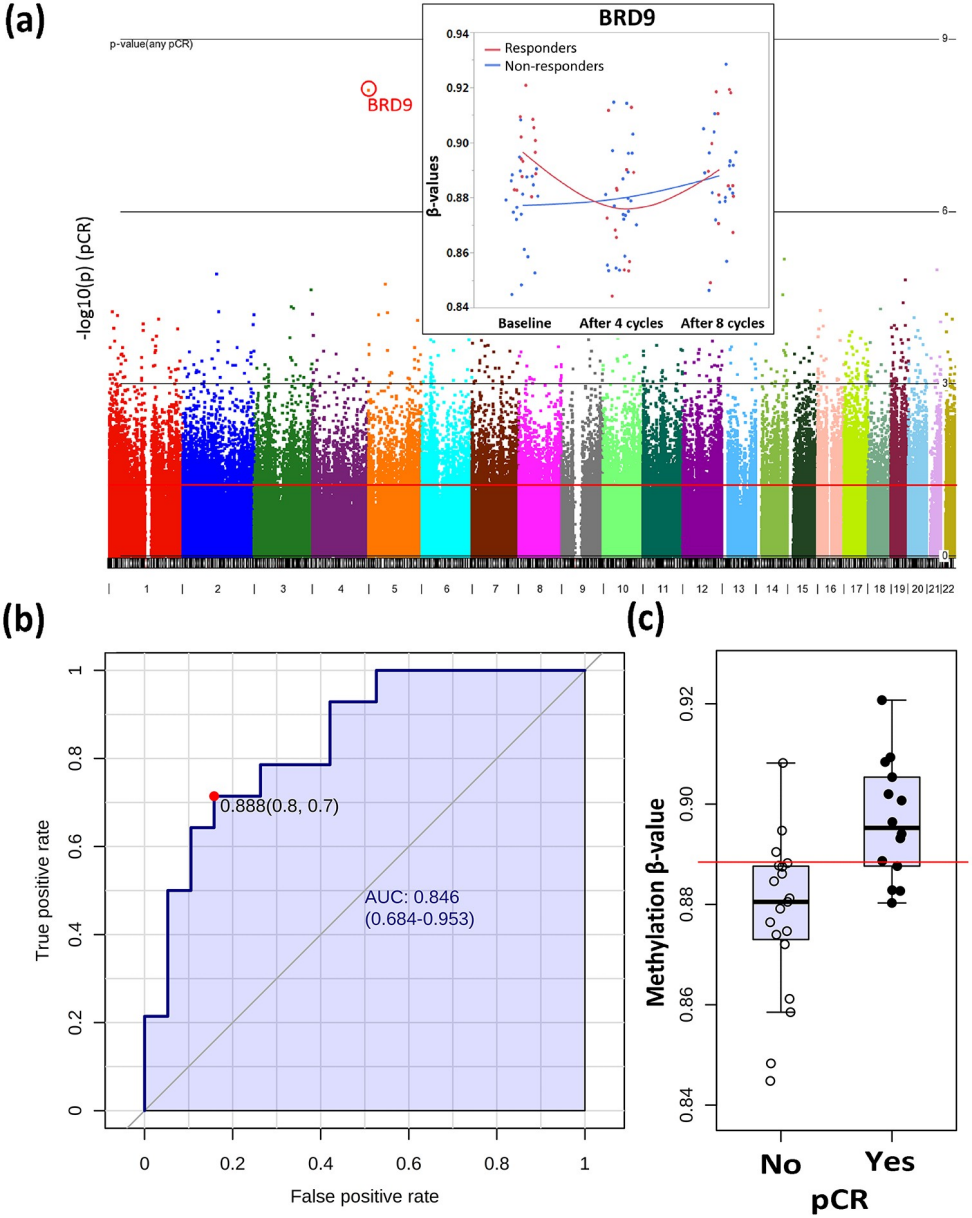
BRD9 (cg06362249) predicts cPR at baseline. (a) Manhattan plot on the chromosomal distribution of differentially methylated CpGs at baseline based on responsiveness to therapy. (b) ROC curve analysis of BRD9. The sensitivity (true positive rate) is on the y-axis, and the specificity (one minus the false positive rate) is on the x-axis with the area under the curve (AUC) of 0.842 (95% CI: 0.708–0.976). (c) Box-plot on the methylation levels of BRD9 between responders (Yes) and non-responders (No). X axis represents β values. A horizontal line in red indicates the optimal cutoff of 0.889 between two groups.

First, we aimed to identify germline DM CpGs at baseline that can serve as non-invasive markers to predict their pCR status following NCT. From our 4-way analysis of variance (ANOVA) model controlling for race, ER status, tumor type, and pCR, 25,321 CpG sites were significantly different between responders and non-responders at baseline (unadjusted *p* < 0.05), but only one CpG passed multiple-testing adjustments (FDR *p* < 0.05, Fig 1a). This CpG site (cg06362249) binds to three *BRD9* transcripts (Chromosome 5, NR_02763, NM_001009877, NM_023924) in the gene body, was hypermethylated among responders at baseline compared to non-responders (Fold change = 1.4; unadjusted *p* = 7.78e-009, FDR *p* = 0.004), decreased its methylation level by 1.24 fold after 4 cycles of treatment among responders (unadjusted *p* = 0.02), but did not change appreciably after treatment among non-responders (Fold change = 1.0; unadjusted *p* = 0.69). The area under the curve (AUC, Fig 1b) for cg06362249 is 0.842 (95%CI: 0.708–0.976) in the Receiver Operating Characteristic (ROC) analysis with the optimal cutoff of 0.889 in β value (% methylation, see Fig 1b and 1c) between responders and non-responders. The methylation levels of cg06362249 were not distinguishable between responders and non-responders after 4 cycles and 8 cycles of treatment (Fig 1a).

### Responder- & non-responder-specific CpG sites

**Fig 2:**
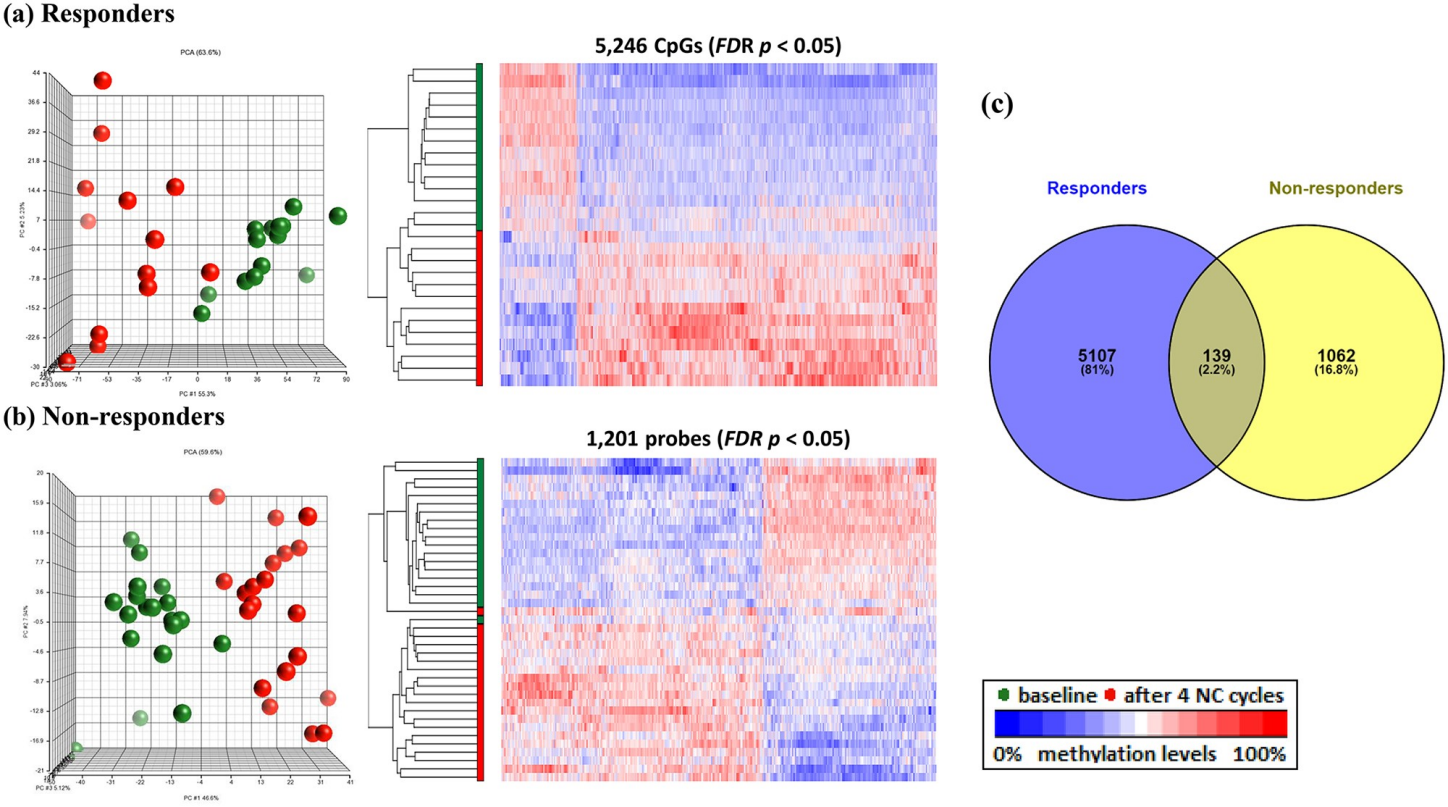
Visualization of the top CpG sites (*FDR p* < 0.05) in principle component analysis and hierarchical clustering distinguishing methylation levels between baseline and after 4 cycles of NCT treatments among responders (a) and non-responders (b). (c) Venn diagram of the DM CpGs showed that 97.8% of DM CpGs were specific to responders or non-responders.

**Fig 3:**
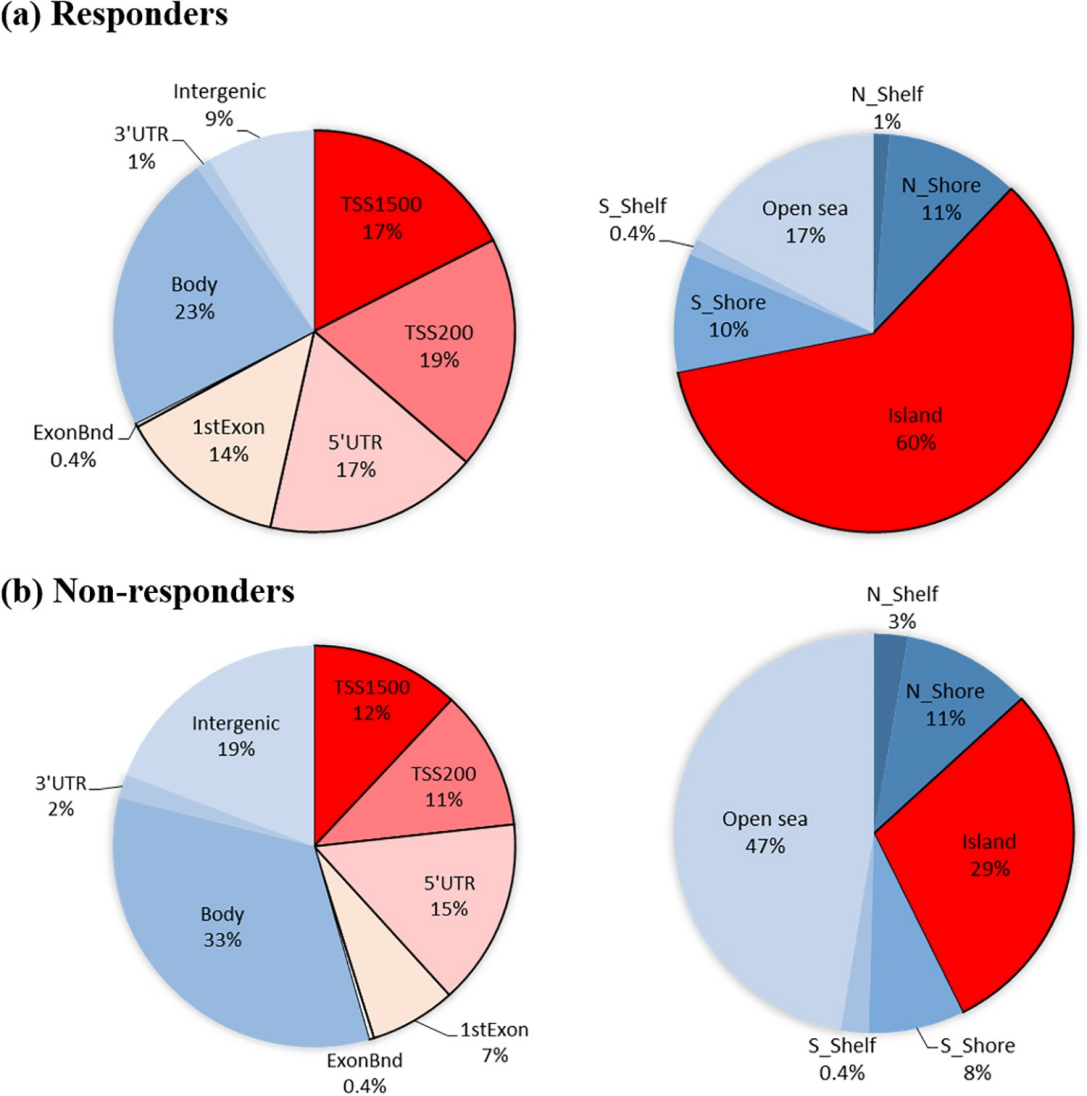
Distribution of differentially methylated CpGs by functional locations (left) and by CpG island regions (right). (a) 67% of DM CpGs among responders were located in the promoter regions that correspond to TSS1500, TSS200, 5’UTR, 1st Exon, and ExonBond, with 60% of CpGs linked to CpG islands, whereas (b) 46% of DM CpGs among non-responders were located in the promoter regions and 29% linked to CpG islands.

To determine the effect of treatment on global methylation profiles specific to the pCR group, separate analyses were performed using the 4-way ANOVA model controlling for race, ER status, treatment cycle, and tumor type for responders and non-responders. There were 5,246 CpGs (FDR *p* < 0.05, Fig 2a) among responders that were DM between baseline and the 4^th^ treatment cycle, 4,984 (95%) of which are projected to be true discoveries under a 5% FDR. A projection of 4,984 true discoveries among responders is slightly higher than this group’s estimate of 4,734 ±22.0 true discoveries from the retrospective power analysis, suggesting that the proportion of CpG sites defined to be “truly positive” (see above) is consistent among responders with the hypothesized rate of 1%. In contrast, there were only 1,201 CpGs (Fig 2b) among non-responders that were DM between baseline and the 4^th^ treatment cycle, 1,141 (95%) of which are projected to be true discoveries under a 5% FDR. A projection of 1,141 true discoveries among non-responders is more than 78% lower that this group’s estimate of 5,222 ±7.2 true discoveries from the retrospective power analysis, indicating that the proportion of “truly positive” CpG sites among non-responders is much lower than the hypothesized rate of 1%. When we inspected the DM CpGs among responders, 67% were localized in the promoter regions that correspond to regions TSS1500 (17%), TSS200 (19%), 5’UTR (17%), 1stExon (14%), and ExonBnd (0.4%) (Fig 3a left). In contrast, 46% of the DM CpGs among non-responders were localized in the promoter regions, including TSS1500 (12%), TSS200 (11%), 5’UTR (15%), 1stExon (7%), and ExonBnd (0.4%) (Fig 3b, left). Further analysis on the distribution of DM CpGs by CpG-island regions revealed more changes in the CpG islands (60%, Fig 3a, right) among responders than the surrounding regions including N Shelf (1%, 4 kb upstream of CpG island), N_Shore (11%, 0–2 kb upstream of the CpG islands), S_Shore (10%, 0–2 kb downstream of the CpG islands), S_Shelf (0.4%, 2–4 kb downstream of the CpG islands), and open sea (17%), while for non-responders, more changes were observed in the open sea (47%, Fig 3b, right) compared to islands (29%). Subsequent analysis using ANOVAs with Bonferroni adjustment selected 19 top CpG sites with the most significant change between baseline and after 4 cycles of NCT treatment, as presented in Table 2. All 10 of the top CpG sites from responders belonged to CpG islands within named genes. In contrast, 8 of the 9 top CpG sites from non-responders belonged instead to “open sea” regions, 3 of which were located in intergenic regions.

**Table 2:** Responder-specific & non-responder specific CpG sites after 4 cycles of treatment.

Probeset ID	UCSC RefGene Name	CHR	Relation to UCSC CpG Island	*Unadjusted p*	*Bonferroni p*	Fold Change*	Methylation level
***Responders-specific CpG sites***							
cg27424117	CDC25B	20	Island	4.2E-09	0.002	-1.4	↑ after treatment
cg25542733	WBSCR27	7	Island	7.3E-09	0.004	-1.7	↑ after treatment
cg13940160	ADNP	20	Island	2.1E-08	0.011	-1.7	↑ after treatment
cg13494334	NEK9	14	Island	2.8E-08	0.015	-1.8	↑ after treatment
cg20161984	COQ2	4	Island	3.0E-08	0.016	-1.3	↑ after treatment
cg04925841	SOX5	12	Island	3.7E-08	0.019	-1.6	↑ after treatment
cg19377384	ACPL2	3	Island	4.9E-08	0.026	-1.5	↑ after treatment
cg05368942	RBM15	1	Island	5.4E-08	0.028	-1.4	↑ after treatment
cg12743270	TLE2	19	Island	5.5E-08	0.029	-1.7	↑ after treatment
cg02709211	RB1CC1	8	Island	6.2E-08	0.033	-1.4	↑ after treatment
***Non-responders-specific CpG sites***							
cg09805347	(Intergenic region)	2	Open sea	1.2E-08	0.006	3.3	↓ *after treatment*
cg17420619	ARHGAP1	11	Open sea	1.6E-08	0.008	-1.4	↑ after treatment
cg26692003	IQSEC1	3	Open sea	1.6E-08	0.009	-3.3	↑ after treatment
cg02402539	(Intergenic region)	19	Open sea	4.2E-08	0.022	-1.4	↑ after treatment
cg26709300	YPEL3	16	N_Shore	5.5E-08	0.029	-1.5	↑ after treatment
cg01680062	RUNX1	21	Open sea	7.1E-08	0.038	2.1	↓ *after treatment*
cg24481263	LINC01258	4	Open sea	8.1E-08	0.043	2.1	↓ *after treatment*
cg00889210	MKL2	16	Open sea	8.8E-08	0.046	-2.3	↑ after treatment
cg17227564	(Intergenic region)	19	Open sea	8.8E-08	0.047	1.5	↓ *after treatment*

*Fold changes represent differences from baseline to the 4^th^ cycles of treatment for each characteristic. Unadjusted *P*-values characterize the statistical significance of the fold changes, while Bonferroni *P*-values are used to select the CpG sites shown in the table. All selected CpG sites have Bonferroni *P* < 0.05.

### Pathway analysis

**Fig 4:**
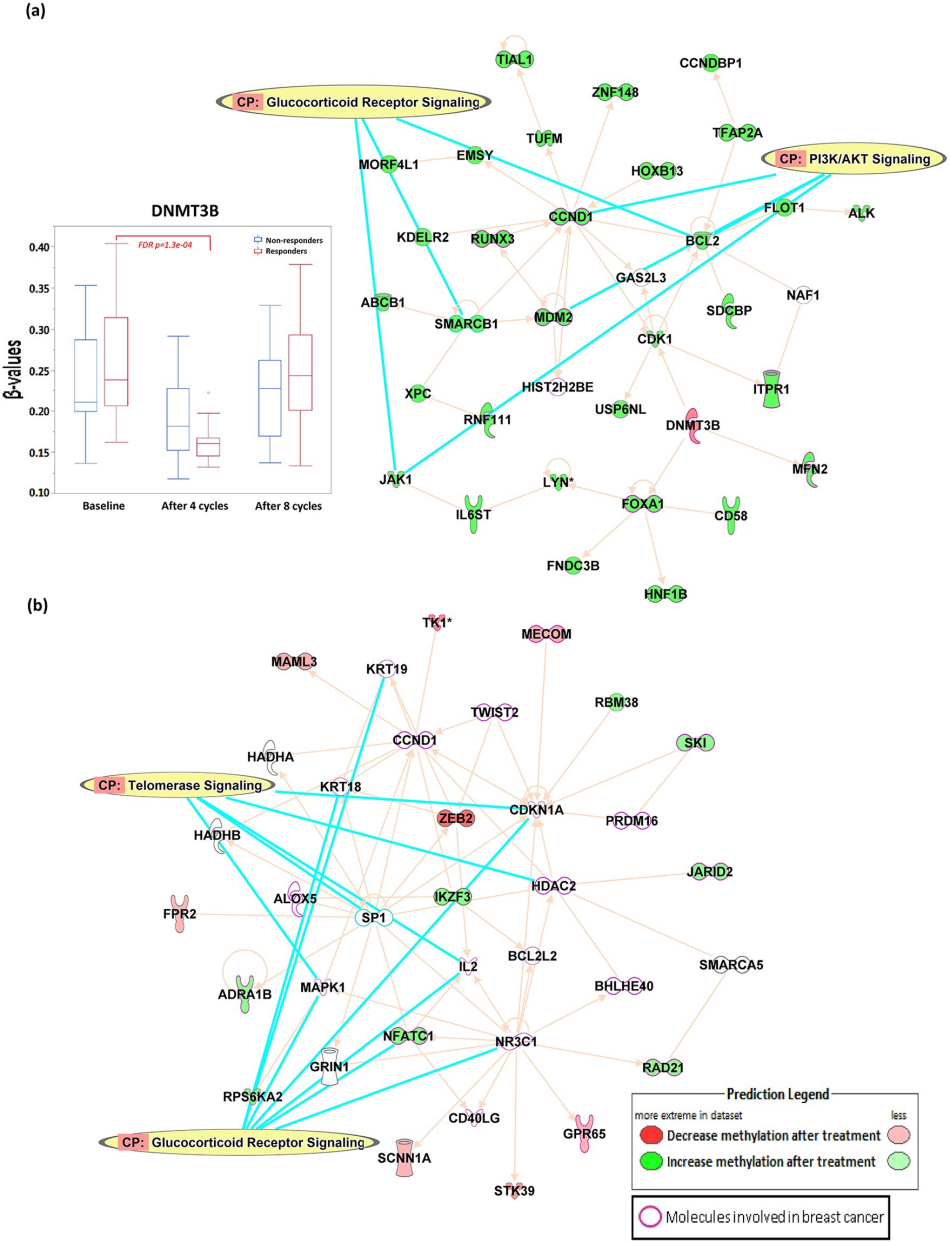
Ingenuity pathway analysis of DM CpGs after 4 cycles of NCT treatment among (a) responders and (b) non-responders.

In order to explore potential molecular events and mechanisms impacted by the neoadjuvant chemotherapy, we applied a statistical-significance threshold of FDR *p* < 0.05 coupled with a scientific-significance threshold of absolute fold change > 1.5 signifying methylation changes of at least 50% from the therapy. This yielded 651 responder-specific DM CpGs and 355 non-responder-specific CpGs (S1 Fig) that were included in separate IPA analyses queried with Ingenuity's knowledge-based networks. Direct relationships focusing on interaction networks observed from all data sources were considered in the analysis, and demonstrated 10 network affected including the top networks affected among responders that were associated with Cellular Development, Cellular Growth and Proliferation, Organismal Development (Score = 49), with 14 molecules involved in breast cancer (*ABCB1*, *ALK*, *BCL2*, *CCND1*, *CDK1*, *DNMT3B*, *FOXA1*, *HIST2H2BE*, *ITPR1*, *JAK1*, *MDM2*, *MFN2*, *RUNX3*, *USP6NL*, Fig 4a). Top canonical pathways involved in the network were PI3K/Akt signaling and glucocorticoid receptor signaling, with *DNMT3B* decreased in methylation levels after 4 cycles of NCT while almost all the other genes in the network were increased in the methylation levels after the therapy (Fig 4a). Among non-responders, the top network out of 20 networks affected was associated with Tissue Morphology, Organismal Development, Embryonic Development (Score = 25) with 23 molecules involved in breast cancer (*ADRA1B*, *ALOX5*, *BCL2L2*, *BHLHE40*, *CCND1*, *CD40LG*, *CDKN1A*, *GPR65*, *HDAC2*, *IL2*, *JARID2*, *KRT18*, *KRT19*, *MAPK1*, *MECOM*, *NFATC1*, *NR3C1*, *PRDM16*, *RAD21*, *RPS6KA2*, *SKI*, *TK1*, *TWIST2*, Fig 4b). Top canonical pathways involved in the network were glucocorticoid receptor signaling and telomerase signaling. Full list of networks affected from both responders and non-responders are available in the S1 and S2 Tables.

## Discussion

Neoadjuvant chemotherapy is administered to patients with operable and locally advanced breast cancers to facilitate breast conservation, or to render inoperable breast tumors viable candidates for surgery. However, the evaluation of clinical response to NCT is delayed until the completion of the therapy. Therefore, biomarkers to predict pCR are urgently needed to avoid unnecessary toxicity to the patient from NCT. Here, we aimed to identify genomic signatures of clinical response among breast cancer patients with very aggressive disease and poor prognosis who underwent NCT using a genome-wide methylation approach. Methylation levels of bromodomain-containing protein 9 (*BRD9*) in the gene body were significantly different at baseline between responders and non-responders, and were the strongest predictors of clinical response in this study. We also identified 5,246 DM CpG sites among responders, with the majority localized in the CpG islands of promoter regions, while 1,201 DM CpG sites were associated with non-responders. The majority of these sites were localized in the intergenic and open-sea regions. Finally, pathway analysis revealed that the DM CpGs among responders were mapped to canonical pathways including PI3K/Akt signaling and glucocorticoid receptor signaling, with decreasing methylation of *DNMT3B*, a major gene responsible for methylation, in responders after 4 cycles of NCT.

BRD9 protein is an epigenetic "reader" of acetylated lysines on post-translationally modified histone proteins that facilitates gene expression. Although little is known about the function of BRD9, copy number gains of the *BRD9* gene in lung cancer patients has been reported and was associated with lung cancer susceptibility [10, 11]. It is over-expressed in cervical cancer [12], promotes *MYC* expression and cell proliferation in acute myeloid leukemia [13]. Protein-engineering and inhibitor studies targeting BRD9 have shown promise in overcoming epigenetically-defined drug resistance [14], and single or combinatorial effects of BRD9 inhibitors with doxorubicin or carboplatin also resulted in anti-proliferative effects in five malignant rhabdoid tumor cell lines [15]. Risdom et al. revealed that epigenetic plasticity mediated by the chromatin modifier BRD4 drives survival of triple-negative breast cancer cells after targeted therapy treatment [16]. In our study, baseline methylation levels of *BRD9* in the gene body were significantly higher among responders compared to non-responders. Gene body methylation has been shown to increase the potential for both somatic and germline mutations [17], and might involve in the recruitment of DNMTs [18, 19]. Functionally, hypermethylation of *BRD9* reduces the amount of available *BRD9* readers, thus preventing recognition of transcription regulators to the acetylated histones, further resulting in the reduced level of transcription. Conversely, lower levels of methylation among non-responders increases the transcription of *BRD9*, thus facilitating gene transcription. Whether the differences affects structural change of the BRD9 gene, binding affinity of the gene, or expression of the gene will need to be further studied.

While chemotherapy profoundly impacts DNA methylation landscape in cancer patients [20, 21], resistance to treatment continues to be a major obstacle for the success of cancer therapy. In our study, increased in the CpG site methylation levels among responders were all mapped to CpG islands including CDC25B, WBSCR27, ADNP, NEK9, COQ2, SOX5, ACPL2, RBM15, TLE2, RB1CC1 (Table 2). Those genes have been shown to have key roles in G2/M cell-cycle progression (CDC25B), methyltransferase (WBSCR27), determination of the cell fate (SOX5), and cell growth (RB1CC1), which the expression levels would expect to be inhibited after treatment. On the other hand, CpG changes among non-responders were mainly mapped to open sea or intergenic regions. Lund et al. reported that the resistance of cisplatin on ovarian cancer cells is associated with loss of hypermethylation in a high number of CpG sites, and are primarily localized in the intergenic regions of the genome [22]. Gene expression can be regulated by the methylation of intergenic regions [23–25], which contain key regulatory elements including enhancers, silencers, and noncoding RNAs. Our results support the role of DNA methylation-associated cancer chemotherapy resistance, and identified putative genes that might be associated with drug resistance. Further investigation in larger studies is needed in order to elucidate the complex molecular mechanisms involved in drug resistance.

Patterns of DNA methylation are established and maintained by DNA methyltransferases (DNMTs) [26]. It is well-known that global DNA hypomethylation in cancer is an early epigenetic abnormality in cancer compared to their normal-tissue counterparts [27, 28], and may result in the accumulation of epigenetic abnormalities leading to chromosomal instability, increased transcription from transposable elements, and an elevated mutation rate due to mitotic recombination [29]. Thus, DNA methylation has been a target of anticancer therapies in the past few decades [30], with much effort being focused on the development of 5-azanucleosides or non-nucleoside DNMT inhibitors to prevent the translation of oncogenic proteins [31, 32]. However, not all breast tumors overexpress DNMTs [33] and little is known about the specificity of DNMTs. In fact, many of the DNMTs are involved in specific and non-specific DNA-methylation machineries [34], suggesting a tight regulation over the DNA methylome to maintain genome and epigenome integrities. Furthermore, DNMT inhibitors have been reported to increase the proliferation of pluripotent stem cell [35] or reprogramming through epigenetic modifications [36], as well as to have immune-suppressive effects [37]. In our study, NCT increased the methylation levels for 4,323 CpG sites and decreased methylation levels of 923 CpGs among responders, potentially through the decreased methylation (increased gene expression) of *DNMT3B* as indicated in Fig 4a, suppressing the expression of certain resistance genes thus leading to pathological complete response. On the other hand, methylation levels of 721 CpGs were increased among non-responders and 480 CpGs were decreased in methylation levels (Fig 2), mostly among the open sea regions. No significant change in methylation was observed for *DNMT3B* among non-responders. Further studies are needed in order to fully understand the function and mechanism underlying the observed differences.

Other attempts at identifying epigenetic markers including targeted approach by examining gene-specific methylation markers including the use of *PITX2* methylation to predict anthracycline-based chemotherapy for triple-negative breast cancer and high-risk breast cancer [38, 39], to examine changes in the methylation levels in estrogen receptor-α promoter regions among triple-negative breast cancer patients [40], as well as global approach to identify HSD17B4 methylation as predictor of HER2-positive breast cancer to trastuzumab and chemotherapy [41]. Another global approach by Gampenrieder et al. reported the use of DNA-methylation signatures in archival formalin-fixed paraffin-embedded (FFPE) specimens to predict Bevacizumab efficacy in HER2-negative metastatic breast cancer by the Illumina Infinium HumanMethylation450 BeadChip [42]. Albeit using the older version of the beadchip, the study identified 9-gene and 3-gene DNA-methylation signatures that were predictive for responders with longer progression-free survival [42]. The study focused on patients with metastatic breast cancers treated with different chemotherapy backbones (taxane or capecitabine) alone or in combination with bevacizumab, which may have different DNA methylation profiles than patients with locally advanced breast cancer treated with neoadjuvant therapy containing bevacizumab in our study. However, their study has a significant age difference (median age 61) compared to ours (median age 43), and did not account for racial differences in the analysis. Since both age [43] and race [44, 45] play important roles in predicting cancer risk, and are related to the DNA-methylation changes in breast cancer tissues [46–48], our study do provide more insights by addressing variability in race, ER status, and tumor type with higher significance cutoff (FDR *p*<0.05, *p*<1.1×10^−9^) and more coverage of CpGs in the new EPIC Beadchips.

To our knowledge, the NIH GDC Data Portal that holds all TCGA database has only data from older chip versions (Illumina Human Methylation 450 and Illumina Human Methylation 27), which does not contain the BRD9 probe we identified from the study. Our samples were collected in 2005–2008 without RNA stabilization reagent so we were unable to validate the results using gene expression assays. However, we do want to acknowledge that the data from Illumina Infinium HumanMethylation BeadChips, especially the Illumina EPIC BeadChips is highly robust and reproducible and is a significant improvement over the HM450 array for human studies [3, 49]. Despite limitations in validation and sample size, this study highlights the use of methylation signatures with the use of stringent statistical methods to correct for multiple comparisons to predict clinical response of breast cancer patients to NCT. In this regard, a larger-scale effort to define the usefulness of the markers may have the potential to greatly expand the clinical utility and to be evaluated in future clinically relevant studies.

## Supporting information

S1 Fig Venn diagram representing DM CpGs from responders and non-responders. (DOCX)

S1 Table Top networks affected from 651 responder-specific CpGs. (XLSX)

S2 Table (XLSX)

## Abbreviations

AA: African American
ANOVA: Analysis of variance
BRD9: Bromodomain Containing 9
CpG: Cytosine-guanine dinucleotide
dbSNP: The Single Nucleotide Polymorphism Database
DCB: Docetaxel, cyclophosphamide and bevacizumab
DM: Differentially methylated
DNMT3A: DNA Methyltransferase 3 Alpha
DNMT3B: DNA Methyltransferase 3 Beta
DNMTs: DNA methyltransferases
EA: European American
ER: Estrogen receptor
FDR: False discovery rate
FFPE: Formalin-fixed paraffin-embedded
HER2: Human epidermal growth factor receptor 2
HSD17B4: Hydroxysteroid 17-Beta Dehydrogenase 4
IPA: Ingenuity Pathway Analysis
IRB: Institutional Review Board
LVEF: Left ventricular ejection fraction
NCBI: National Center for Biotechnology Information
NCT: Neoadjuvant chemotherapy
PCA: Principal Component Analysis
pCR: Pathologic complete response
PITX2: Paired Like Homeodomain 2
ROC: Receiver Operating Characteristic
SNPs: Single-nucleotide polymorphisms
SWAN: Sub set-quantile Within Array Normalization
