# Supplementary material for: Genome-wide DNA methylation signatures to predict pathologic complete response from combined neoadjuvant chemotherapy with bevacizumab in breast cancer
Source: PLoS One. 2020 Apr 16;15(4):e0230248. doi: 10.1371/journal.pone.0230248 (PMC7162481; doi:10.1371/journal.pone.0230248)
Supplement: S1 Fig — (DOCX) [file pone.0230248.s001.docx]

# **Supplemental figure**


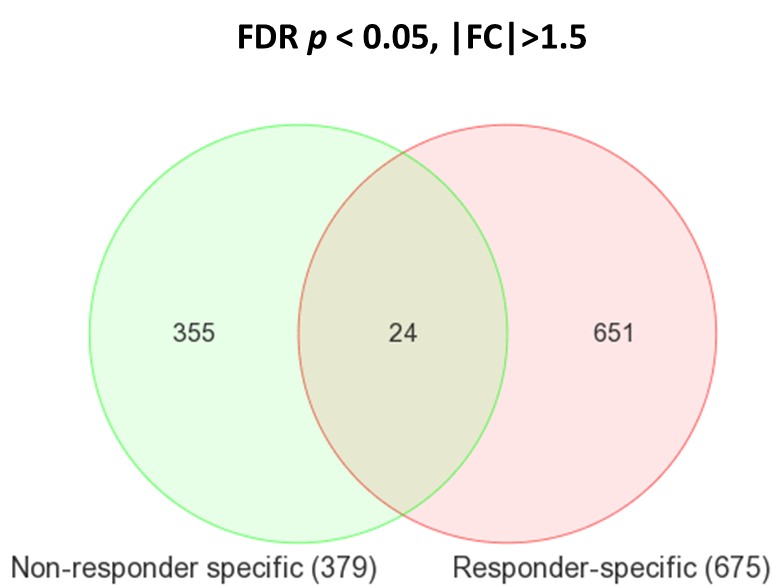


## S1 Fig. Venn diagram representing DM CpGs from responders and non-responders.
